# Determinants of health-related quality of life in recently detoxified patients with severe alcohol use disorder

**DOI:** 10.1186/s12955-022-02058-x

**Published:** 2022-10-31

**Authors:** Najlaa Lahbairi, Alice Laniepce, Shailendra Segobin, Nicolas Cabé, Céline Boudehent, François Vabret, Géraldine Rauchs, Anne-Lise Pitel

**Affiliations:** 1 Neuropsychologie et Imagerie de la Mémoire Humaine, Normandie Univ, UNICAEN, PSL Université de Paris, EPHE, INSERM, U1077, CHU de Caen, GIP Cyceron, 14000 Caen, France; 2 Service d’Addictologie, Centre Hospitalier Universitaire de Caen, 14000 Caen, France; 3 Normandie Univ, UNICAEN, INSERM, U1237, PhIND Physiopathology and Imaging of Neurological Disorders, NEUROPRESAGE Team, (Institut Blood and Brain @ Caen-Normandie), Cyceron, 14000 Caen, France; 4 Normandie Univ, UNIROUEN, CRFDP (EA 7475), 76000 Rouen, France

**Keywords:** Health-related quality of life, Alcohol use disorder, Cognition, Sleep, Anxiety, Impulsivity

## Abstract

**Background:**

Health-related quality of life (HRQoL) is an important clinical outcome in Alcohol Use Disorder (AUD) and is considered as a relevant indicator of treatment success. While a better understanding of the factors affecting HRQoL would enable to adjust patients’ care to favour treatment outcome, the determinants of HRQoL in AUD remain unclear. This study aims at describing HRQoL in AUD patients and at identifying its best predictors.

**Methods:**

53 recently detoxified patients with severe AUD (sAUD) underwent a cognitive assessment and filled in a HRQoL questionnaire dedicated to AUD patients (Alcohol Quality of Life Scale; AQoLS), as well as questionnaires concerning socio-demographics, alcohol history, sleep quality, depression, anxiety and impulsivity. 38 healthy controls (HC) underwent the same assessment (except AQoLS) in order to identify the altered cognitive and clinical variables that could potentially be determinants of HRQoL in sAUD.

**Results:**

sAUD patients reported that alcohol affects their HRQoL mainly in the “negative emotions”, “control”, “relationships”, and “sleep” domains. Compared to HC, they were impaired on episodic memory, working memory, executive functions, and processing speed tasks. They also reported lower sleep quality, higher depression, anxiety and impulsivity. No association was found between AQoLS total score and socio-demographics, cognitive performance, or sleep quality in patients. We found a significant correlation between HRQoL and depression/anxiety as well as impulsivity. Anxiety and impulsivity were indeed the only significant predictors of HRQoL, explaining 47.7% of the variance.

**Conclusion:**

Anxiety and impulsivity are crucial determinants of HRQoL in recently detoxified sAUD patients. Since anxiety and impulsivity are frequent issues in addiction and especially in AUD, they should be particularly considered by clinicians to favour treatment outcomes.

## Introduction

Alcohol Use Disorder (AUD) is among the most prevalent mental disorders worldwide [1]. According to the World Health Organization’s global status report on alcohol and health [2], an estimated 283 million people has AUD, with Europe showing the highest prevalence (8.8% of the adult population) followed by the US (8.2% of the adult population). Abstinence has long been considered as the only final therapeutic goal for AUD [3]. However, this clinical approach has a number of limitations: (i) only a few AUD patients seek treatment [4], (ii) among those who seek treatment, many patients report that they are not ready to completely stop drinking [5] and the risk of relapse remains high [6] even after a long period of abstinence [7], and (iii) quantitative criteria based on the amount of alcohol consumed or the length of the abstinence period seem to be a weak indicator to evaluate the effectiveness of a treatment [8]. For all these reasons, reducing alcohol consumption without necessarily aiming to achieve total abstinence is gaining an increasing interest in clinical practice. This novel strategy gives a crucial place to qualitative assessments. In this perspective, quality of life (QoL) has emerged as a new treatment outcome that is more adapted to patients’ concerns. Indeed, QoL is considered to be not only a measure of treatment effectiveness [8] but also a motivational tool [9]. Thus, improving QoL might be a primary endpoint in the management of AUD.

QoL is defined as “an individual’s perception of his/her position in life, and in the context of culture and value system in which he/she lives, and also in relation to his/her goals, expectations, standards, and concerns” [10]. In a biomedical context, the term health-related quality of life (HRQoL) has been proposed [11], referring to “the patient’s subjective perception of the impact of his/her disease and its treatment(s) on his/her daily life, physical, psychological and social functioning and well-being [12]. HRQoL is known to be more robust, highly sensitive to change [10] and more relevant for evaluating the effectiveness of a treatment than QoL in AUD [13]. QoL differs from HRQoL since it investigates the overall quality of life irrespectively of any health condition, whereas HRQoL necessarily refers to the impact of a particular health status. Nevertheless, the two terms are often used interchangeably in the literature, leading sometimes to confusion [14].

As HRQoL is considered as a relevant measure of treatment outcome, several studies have been conducted in various conditions to identify its predictors. These studies reported that cognitive impairments, sleep disturbances, mood disorders, impulsivity and demographic variables such as age, education, and gender were significantly related to HRQoL in multiple sclerosis, cancer and chronic diseases in elderly people [15–18]. In AUD, general QoL seems to be associated with gender, age (19, 20), depression [11], and severity of AUD [19]. But to date, no study has examined the specific determinants of HRQoL in AUD.

Some studies revealed that HRQoL is altered in AUD patients [11]. However, despite the fact that HRQoL is considered crucial in the management of AUD, it remains little explored and only with generic scales, not specific to AUD [21]. Because these scales were designed by clinicians or researchers, they did not include opinions and perceptions of patients, contrary to the recommendations of the Food and Drug Administration [22] that encourages to use patient-reported outcome (PRO) measures.

The only specific HRQoL assessments in AUD was the AlQoL-9 [23]. However, this scale was derived from a generic scale, called the SF-36, by eliminating non relevant items. In this respect, this tool did not meet the criteria for PRO instruments, which are defined as reports that came directly from the patient without amendment or interpretation of the patient’s response by a clinician or anyone else [22]. To remedy this, Luquiens et al. [13] [24] have developed a specific tool, called “Alcohol Quality of Life Scale” (AQoLS), designed from the patients’ perspective. The overall goal of the present study is to investigate HRQoL in recently detoxified patients with severe AUD (sAUD) using this tool. We first aimed at providing a better description of HRQoL in sAUD by highlighting the most impacted domains. We also aimed at exploring the relationships between HRQoL and socio-demographic, cognitive and clinical variables altered in sAUD compared with healthy controls (HC) to identify the most significant determinants of HRQoL in sAUD.

## Materials and methods

### Participants

We conducted an observational study enrolling 53 recently detoxified sAUD inpatients. None of them had a history of neurological, endocrinal, or infectious diseases, neither depression assessed using both the Beck Depression Inventory (BDI) [25] and a psychiatric assessment, nor other forms of substance use disorder (except tobacco). All participants were informed about the study approved by the local ethics committee of Caen University Hospital (CPP Nord Ouest III, no. IDRCB: 2011-A00495-36) before their inclusion and signed a written informed consent form.

sAUD patients were recruited by clinicians while they were receiving withdrawal treatment as inpatients at Caen University Hospital. Although recently detoxified, patients no longer showed signs of withdrawal at inclusion as assessed by the Cushman’s scale [26]. sAUD patients met “alcohol dependence” criteria according to the DSM-IV-TR [27] or “severe AUD” criteria according to the DSM-5 [28] for at least five years.

We also recruited 38 healthy controls (HC) in order to highlight, in sAUD, the altered cognitive and clinical factors that could be tested as determinants of HRQoL.

sAUD patients and HC subjects were matched for age, gender and education (p = 0.33, p = 0.21 and p = 0.12 respectively). Patients tended to live more frequently alone than HC (p = 0.05) (Table 1**)**. The protocol was conducted at the Addiction department for sAUD patients and in the laboratory for HC.

**Table 1:** Socio-demographic, cognitive and clinical features of the severe Alcohol Use Disorder (sAUD) patients and Healthy Controls (HC).

	sAUD patients (n = 53) (M ± SD)	HC subjects (n = 38) (M ± SD)	Between-group comparisons
**SOCIO-DEMOGRAPHIC**			
Age (years)	46.15 ± 10.13	44.34 ± 6.07	p = 0.33
Gender, men (%)	86.79%	94.73%	p = 0.21
Education (years)	11.32 ± 2.09	11.82 ± 0.73	p = 0.12
Living status, living alone (%)	54.72%	34.21%	p = 0.05
**COGNITIVE**			
Episodic memory (z-score)	-1.55 ± 1.22	0 ± 1	HC > sAUD^*^
Working memory (z-score)	-1.27 ± 0.82	0 ± 1	HC > sAUD^*^
Executive functions (z-score)	-1.08 ± 1.49	0 ± 1	HC > sAUD^*^
Processing speed (z-score)	-1.57 ± 1.65	0 ± 1	HC > sAUD^*^
**CLINICAL**			
**Sleep**			
PSQI	8.74 ± 3.27 (2MD)	2.37 ± 1.51	HC < sAUD^*^
**Depression and anxiety**			
BDI	17.30 ± 10.91 (1MD)	2.89 ± 3.09	HC < sAUD^*^
STAI B (trait anxiety)	51.21 ± 10.62	32.16 ± 6.87	HC < sAUD^*^
**Impulsivity**			
S-UPPS-P	48.45 ± 10.99	33.24 ± 11.15	HC < sAUD^*^
Negative Urgency	10.38 ± 3.41	7.13 ± 3.23	HC < sAUD^*^
Lack of Premeditation	8.98 ± 2.54	6.24 ± 2.11	HC < sAUD^*^
Lack of perseverance	8.32 ± 2.92	5.68 ± 1.95	HC < sAUD^*^
Sensation Seeking	10.64 ± 8.04	6.79 ± 2.36	HC < sAUD^*^
Positive Urgency	11.43 ± 2.94	7.5 ± 3.35	HC < sAUD^*^
**Alcohol history**			
AUDIT	28.58 ± 5.75	2.42 ± 1.64	HC < sAUD^*^
Age of onset of AUD (years)	31.30 ± 8.87 (3MD)	/	/
Daily alcohol consumption during the month preceding withdrawal (units ^a^)	19.30 ± 7.01 (1MD)	/	/

AUD: Alcohol Use Disorder; n: sample size; M: mean; SD: standard deviation; HC: healthy controls; BDI: Beck Depression Inventory; STAI: State-Trait Anxiety Inventory; AUDIT: Alcohol Use Disorders Identification Test; PSQI: Pittsburgh Sleep Quality Index; S-UPPS-P: Short form of negative Urgency, lack of Premeditation, lack of Perseverance, sensation Seeking, Positive urgency. MD: Missing Data; ^*^: p < 0.01; ^a^: an alcohol unit = 10 g of pure alcohol

HC subjects were interviewed with the Alcohol Use Disorder Identification Test (AUDIT) [29] to ensure that they did not meet the criteria for alcohol abuse (AUDIT < 7 for men and < 6 for women). None of the controls had a BDI score > 29 [25] nor sleep complaint (Pittsburgh Sleep Quality Index [PSQI] score ≤ 5) [30].

### Clinical and cognitive assessments

#### Assessment of Health-Related Quality of Life (HRQoL) in sAUD patients

HRQoL was assessed using AQoLS, which is a specific self-assessment questionnaire including 34 items and measuring the specific impact of alcohol on HRQoL over the last 4 weeks. AQoLS has been specifically developed for AUD patients as all the items have been directly generated by patients and reflect, therefore, their concerns. AQoLS explores 7 domains: activities, relationships, living conditions, negative emotions, self-esteem, control and sleep. The number of items for each domain and some examples are presented in Table 2. For each item, the level of agreement is reported based on a 4-point Likert scale, ranging from 0 (not at all) to 3 (very much). Thus, a high total AQoLS score reflects poor HRQoL.

**Table 2:** AQoLS domains, number of items and examples

AQoLS domains	Number of items	Examples	
Activities	10	I have felt I miss out on everyday activities with family and friends	
Relationships	6	Alcohol has interfered with my relationships with friends	
Living conditions	4	Alcohol has had a negative effect on my housing situation	
Negative emotions	2	I have worried about alcohol causing problems in my life	
Self-esteem	5	I have neglected my general health	
Control	5	I have planned my days around alcohol	
Sleep	2	I have not been getting enough sleep	

AQoLS: Alcohol Quality of Life Scale

#### Neuropsychological assessment

Participants underwent a detailed neuropsychological examination targeting verbal episodic memory, working memory, executive functions, and processing speed.

##### Episodic memory

Verbal episodic memory was assessed using the French version of the Free and Cued Selective Reminding Test (FCSRT) [31]. We used the sum of the three free recalls of learning trials.

##### Working memory

Verbal working memory was assessed with the digit span tasks (forward and backward) of WAIS III [32].

##### Executive functions

We evaluated mental flexibility using the number of perseverative errors on the Modified Card Sorting Tests (MCST) [33]. Inhibition was measured using the time (in seconds) needed to complete the interference condition minus the time needed for the denomination condition of the Stroop Test [34].

##### Processing speed

Processing speed was assessed using the denomination condition of the Stroop Test (time in seconds) [34].

#### Assessment of subjective sleep quality

Subjective sleep quality was assessed using the PSQI, which is a 19-item questionnaire exploring sleep quality and sleep disturbances. Seven components are explored (subjective sleep quality, sleep latency, sleep duration, habitual sleep efficiency, sleep disturbances, use of sleep medications, and daytime dysfunction) and scored on a scale ranging from 0 to 3. The PSQI total score corresponds to the sum of the scores obtained for each component, and ranges from 0 (no difficulty) to 21 (major sleep difficulties).

Of note, the original version of the PSQI questioning the previous month has been proposed to HC subjects and 11 sAUD patients. A modified version assessing sleep quality during the previous week (made with the authors’ agreement) was administered to 20 sAUD patients (two patients had missing data). Since a comparison between the original and modified versions of the PSQI did not reveal any significant difference (t (49) = 1.04, p = 0.30), PSQI data were pooled together.

#### Assessment of mood

Participants completed the BDI [25], a 21-item self-reported questionnaire that evaluates symptoms and overt behavioural manifestations of depression. Each item has four possible responses, ranging from 0 (e.g. “I do not feel sad”) to 3 (e.g. “I am so sad or unhappy that I can’t stand it”). The total score ranges from 0 to 63 with higher scores indicating higher levels of depression.

Participants also filled out the STAI B [35], a 20-item self-completed questionnaire that measures trait anxiety defined as the propensity to be generally anxious. Each item has four possible responses ranging from 1 (almost never) to 4 (almost always): a higher score indicates greater trait anxiety.

#### Assessment of impulsivity

Participants completed the short version of the Urgency, Premeditation, Perseverance, Sensation Seeking, and Positive Urgency impulsivity behavioral scale [36], which is a 20-item self-reported questionnaire that measures personality facets associated with impulsivity. The S-UPPS-P includes five subscales (*negative urgency*: tendency to act rashly under extreme negative emotions, *lack of premeditation*: tendency to act without thinking, *lack of perseverance*: inability to remain focused on a task, *sensation seeking*: tendency to seek out novel and thrilling experiences, and *positive urgency*: tendency to act rashly under extreme positive emotions). Each item of the S-UPPS-P has four possible responses ranging from 1 (strongly agree) to 4 (strongly disagree) with higher scores indicating a higher level of impulsivity.

#### Alcohol history

Alcohol use was first explored using the AUDIT [29]. The total score ranges from 0 to 40 with higher scores indicating greater hazardous drinking. We also collected the age of onset of AUD, and daily alcohol consumption during the month preceding alcohol withdrawal.

### Statistical analyses

Cognitive data were converted into standardized *z-scores* using the mean and standard deviation of the HC. When necessary, the direction of the *z-score* was reversed (e.g. number of errors) so that all the z-scores had the same direction: the higher the *z-score*, the better the performance. When a cognitive domain included several variables (e.g. executive functions), a composite score was calculated by averaging the z-scores obtained for each variable.

We first used descriptive statistics to analyze HRQoL (AQoLS total score and scores for each domain) in sAUD patients. Since the domains of the AQoLS do not have the same number of items (see Table 2**)**, we normalized the subscores, dividing, for each patient, the score of each domain by the maximum score that could be obtained for that domain. Thus, for each domain the score ranges from 0 to 1. Then, in order to compare all the domains to each other, repeated measures analysis of variances (ANOVA) were conducted followed by Bonferroni *post hoc* tests.

Then, to identify altered cognitive and clinical variables that could be potential determinants of HRQoL in sAUD, Student’s *t*-tests were carried out to compare sAUD patients and HC on these variables.

In sAUD patients, the relationships between HRQoL and socio-demographic (age and education), cognitive and clinical variables were examined using Pearson’s correlations. Student’s *t*-tests were performed to examine a potential effect of gender and living status (alone vs. with a partner) on HRQoL. Variables that were significantly correlated with AQoLS were entered in a stepwise linear regression analysis to determine the best predictor(s) of HRQoL. Both, backward and forward models were used to ensure congruence.

An exploratory analysis was also performed to examine the relationships between the different AQoLS domains and socio-demographic, cognitive, and clinical variables. For this analysis, we notably used the scores on the subscales of the S-UPPS-P questionnaire.

Given the number of statistical analyses, the threshold of statistical significance was set at p < 0.01 for all analyses. Statistical analyses were carried out using JASP (version 0.13.1).

## RESULTS

### HRQoL in sAUD patients

The mean AQoLS score (± standard deviation) was 49.94 ± 18.25 with a great variability between patients (total score ranging from 18 to 92). The scores obtained for each AQoLS domain as well as the total score are presented in Table 3.

**Table 3:** Results on the AQoLS questionnaire in sAUD patients (raw data)

	Activities	Relationships	Living conditions	Negative emotions	Self-esteem	Control	Sleep	AQoLS Total score
**Mean**	12.19	10.62	4.45	3.70	6.70	7.06	2.23	49.94
**Standard Deviation**	5.87	3.74	2.65	1.61	3.57	3.84	2.04	18.25
**Range**	3–26	3–18	1–10	0–6	0–15	1–15	0–6	18–92
**Maximum possible score**	30	18	12	6	15	15	6	102

AQoLS: Alcohol Quality of Life Scale, sAUD: severe Alcohol Use Disorder

**Fig. 1:**
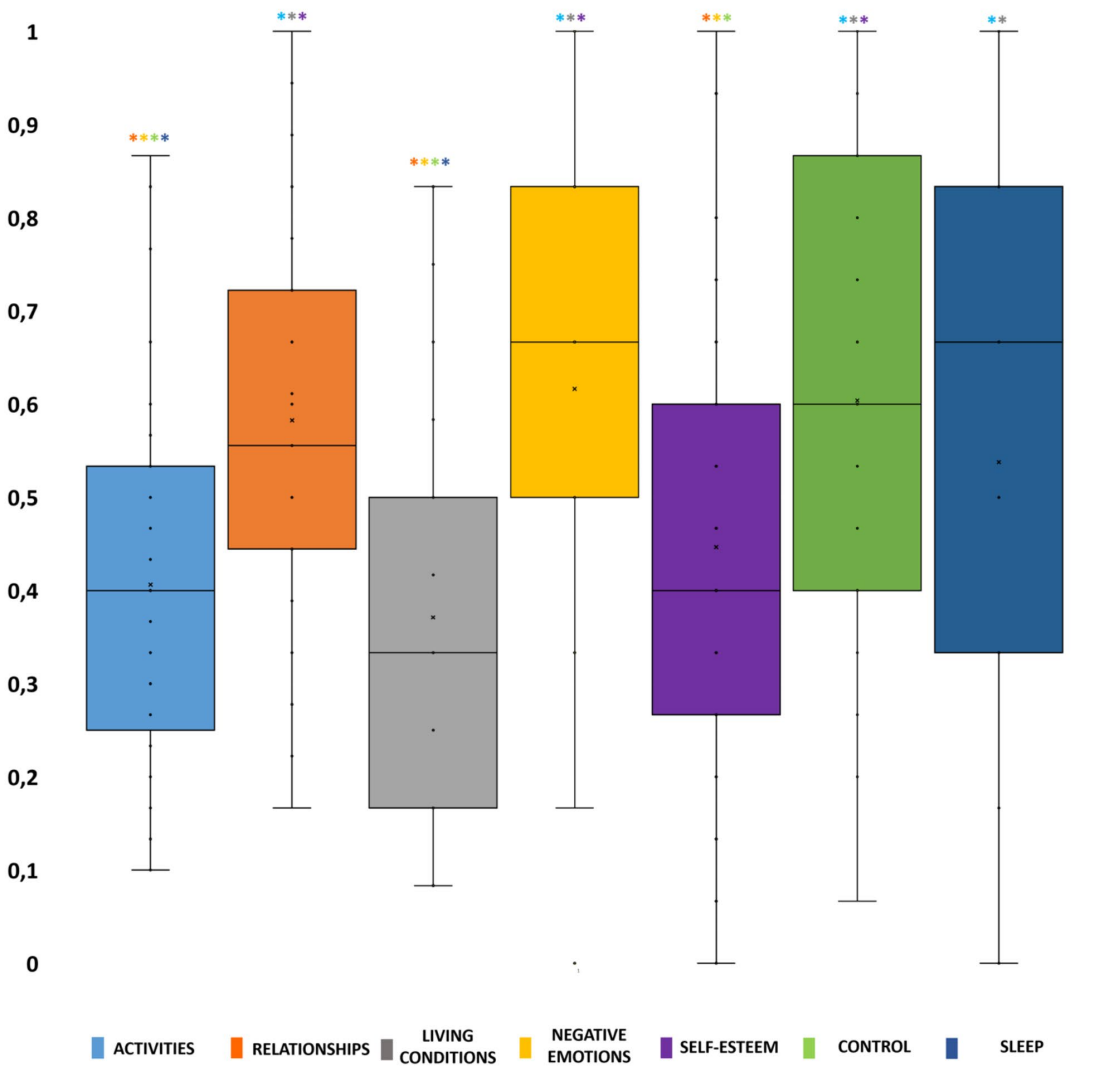
**Normalized domains of HRQoL in recently detoxified sAUD patients** For each boxplot, the median is represented by the bold line and the mean is represented by “x” *: significant difference compared to another domain (p < 0.01) using the specific color of each boxplot

As presented in Fig. 1, we identified significant statistical differences between a set of AQoLS domains.

Indeed, after normalization, the four main domains for which sAUD patients complained the most (namely “negative emotions”, “control”, “relationships” and “sleep”) (Table 4) were all significantly different from the following domains “activities”, “living conditions”, and “self-esteem” for which patients complained the least. In addition, the score for the “sleep” domain was significantly higher compared to those obtained in the “activities” and “living conditions” domains (see Fig. 1).

**Table 4:** Results on the AQoLS questionnaire in sAUD patients (normalized data)

	Activities	Relationships	Living conditions	Negative emotions	Self-esteem	Control	Sleep
**Mean**	0.41	0.58	0.37	0.62	0.45	0.60	0.54
**Standard Deviation**	0.20	0.20	0.22	0.27	0.24	0.26	0.34
**Range**	0.10–0.87	0.17–1	0.08–0.83	0–1	0–1	0.07–1	0–1

AQoLS: Alcohol Quality of Life Scale, sAUD: severe Alcohol Use Disorder

### Comparison of neuropsychological performance, sleep, mood and impulsivity between HC and sAUD patients

sAUD patients presented lower performance than HC for all the cognitive functions assessed (episodic memory: t (89) = -6.43, p < 0.001; working memory: t (89) = -7.96, p < 0.001; executive functions: t (89) = -4.11, p < 0.001 and processing speed: t (89) = -5.21, p < 0.001; Table 1).

The PSQI score was higher in sAUD patients than in HC (t (87) = 11.14, p < 0.001; Table 1), reflecting more severe sleep disturbances.

Compared to HC, sAUD patients exhibited higher BDI and STAI B scores (t (88) = 7.91, p < 0.001 and t (89) = 9.69, p < 0.001 respectively; Table 1), indicating that sAUD patients had more depressive symptoms and were more anxious than HC.

A significant difference was observed between the two groups on the S-UPPS-P. Compared to HC, sAUD patients showed higher scores on the S-UPPS-P total score (t (89) = 6.47, p < 0.001), reflecting greater impulsivity. More precisely, sAUD patients showed higher scores than HC on all the five subscales of the S-UPPS-P: negative urgency (t (89) = 4.63, p < 0.001), lack of premeditation (t (89) = 5.45, p < 0.001), lack of perseverance (t (89) = 4.84, p < 0.001, sensation seeking (t (89) = 2.85, p = 0.005), and positive urgency (t (89) = 5.93, p < 0.001).

### Determinants of HRQoL in sAUD patients

No significant correlation was found between the AQoLS total score and the demographic variables in sAUD patients (age: r = -0.21; education: r = 0.09, all p values > 0.13).

Student t-tests did not reveal any significant effect of gender (t (51) = 0.82, p = 0.41) or living status (t (51) = -0.19, p = 0.85) on the AQoLS total score.

The AQoLS total score did not correlate with cognitive performance either (episodic memory: r = 0.03; working memory: r = -0.07; executive functions: r = 0.25; processing speed: r = 0.22; all p values > 0.06).

None of the variables reflecting sleep disturbances (PSQI: r = 0.22, p = 0.12) or alcohol history (AUDIT: r = 0.27, p = 0.05; age of onset of AUD: r = -0.23, p = 0.11; daily alcohol consumption: r = 0.17, p = 0.22) were significantly related to the AQoLS total score.

**Fig. 2:**
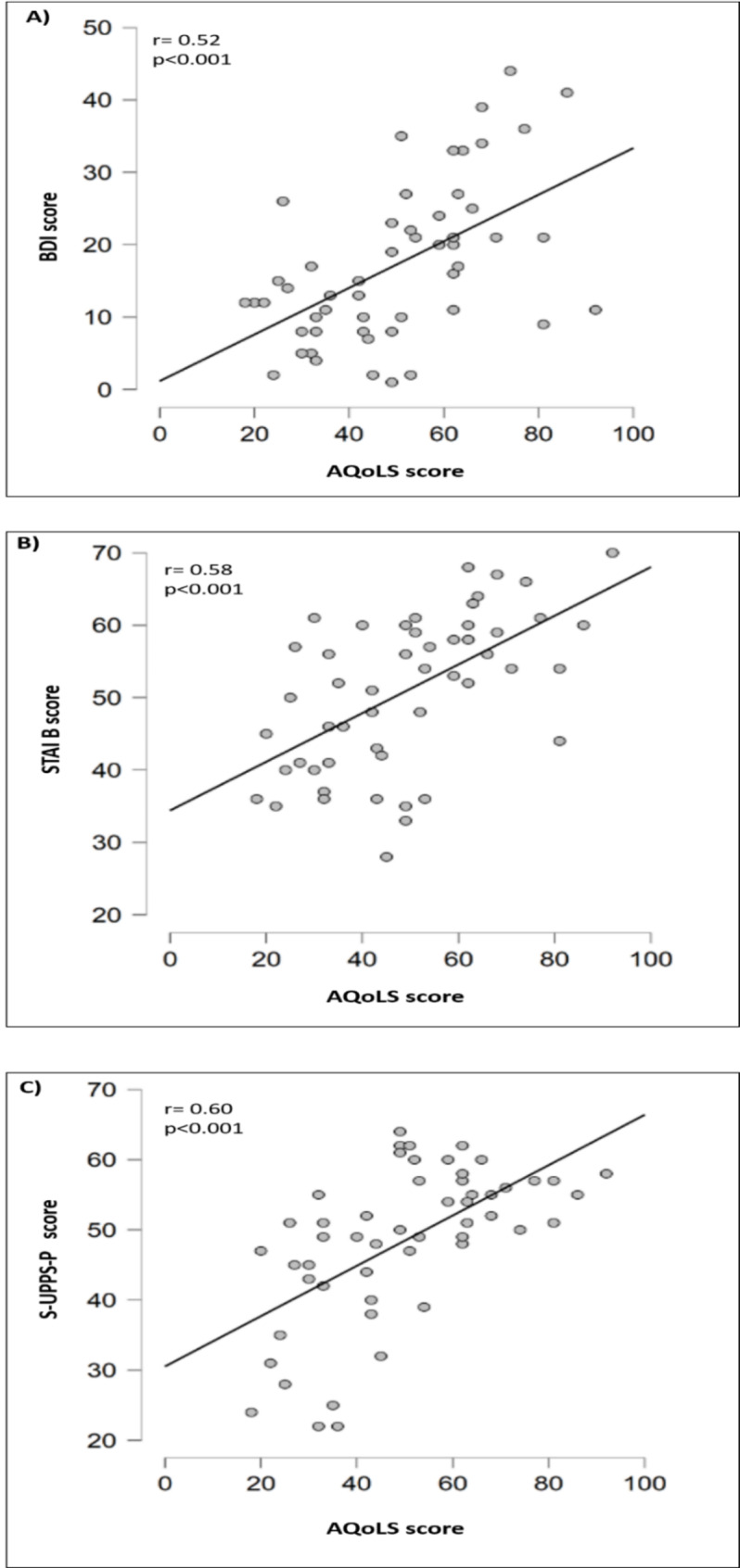
**Relationships between HRQoL and (A) depression, (B) anxiety, and (C) impulsivity in recently detoxified sAUD patients** AQoLS: Alcohol Quality of Life Scale; BDI: Beck Depression Inventory; STAI B: State-Trait Anxiety Inventory part B; S-UPPS-P: Short form of- negative Urgency, lack of Premeditation, lack of Perseverance, sensation Seeking, Positive urgency. r: Person’s correlation coefficient, *p < 0.01

The BDI as well as the STAI B scores show a significant positive correlation with the AQoLS total score (r = 0.54, r = 0.58 respectively; p < 0.001, Fig. 2A and B). Thus, the more depressed and anxious sAUD patients were, the poorer was their HRQoL. The S-UPPS-P total score (r = 0.60, p < 0.001) positively correlated with the AQoLS total score (Fig. 2C). Thus, the more sAUD patients exhibited impulsive behaviours, the poorer was their HRQoL.

A stepwise multiple linear regression analysis was then conducted using the AQoLS total score as the dependent variable and the BDI, STAI-B and S-UPPS-S total scores as predictors based on the results of the correlation analyses. Since the backward and the forward models provided the same results, we present only the forward model. The first step showed that the S-UPPS-P score explained 35.8% of the variance of the AQoLS total score. In a second (and final) step, the model showed that the S-UPPS-P and the STAI B scores were the only significant predictors of the AQoLS total score, explaining 47.7% of the variance (Table 5).

**Table 5:** Results of forward stepwise regressions models showing the variables most strongly associated with HRQoL in sAUD patients

Factor	Unstandardized coefficient (95% CI)	β*	R^2^	*p* value
**Step 1**				
Intercept	2.17 (-16.56–20.90)	NA	NA	0.82
S-UPPS-P score	0.99 (0.61–1.37)	0.60	0.358	< 0.001
**Step 2**				
Intercept	-17.21 (-37.89–3.47)	NA	NA	0.10
S-UPPS-P score	0.67 (0.28–1.07)	0.406	NA	0.001
STAI B score	0.68 (0.27–1.09)	0.395	0.445	0.002
Full model	NA	NA	0.477	< 0.001

CI: Confidence Interval, β*: Standardized beta coefficients, R^2^: R-Squared, NA: Not Applicable

S-UPPS-P: Short form of- negative Urgency, lack of Premeditation, lack of Perseverance, sensation Seeking, Positive urgency; STAI B: State-Trait Anxiety Inventory part B

### Exploratory analysis

Using the scores of the different AQoLS domains, we found that the scores for the “control” (r = 0.47, r = 0.62), “activities” (r = 0.55; r = 0.59), “self-esteem” (r = 0.56; r = 0.56), “sleep” (r = 0.47; r = 0.53) domains significantly correlated with the STAI B and the S-UPPS-P scores (all p values < 0.001). This indicates that the more the sAUD patients exhibited impulsive behaviors, the more they perceived their control, activities, self-esteem, sleep as being impacted by alcohol consumption. Moreover, we found that the scores on “living conditions” and “negative emotions” domains significantly correlated with the STAI B and the S-UPPS scores (r = 0.39; r = 0.40 respectively, all p values < 0.004). Thus, the more sAUD patients were anxious, the poorer were their living conditions. Furthermore, the more patients exhibited impulsive behaviors, the more they expressed negative emotions.

When considering the scores on the different subscales of the S-UPPS-P, we found significant relationships between positive urgency and the “activities” (r = 0.45, p < 0.001), “negative emotions” (r = 0.36, p = 0.008), “self-esteem” (r = 0.43, p < 0.001), “control” (r = 0.58, p < 0.001) and “sleep” domains (r = 0.11, p < 0.001). The lack of premeditation significantly correlated with the “activities’, “self-esteem”, “control” and “sleep” domains (r = 0.58; r = 0.46; r = 0.52; r = 0.46 respectively, all p values < 0.001). Significant relationships were also found between the lack of perseverance and the “activities” (r = 0.39, p = 0.004) and “control” domains (r = 0.37, p = 0.006). Finally, negative urgency significantly correlated with the “self-esteem” (r = 0.37, p = 0.006), “control” (r = 0.48, p < 0.001) and “sleep” domains (r = 0.37, p = 0.007).

## Discussion

Our study aimed at describing HRQoL in recently detoxified sAUD patients using a tool specifically designed for this clinical population. We also investigated the relationships between HRQoL and socio-demographic, cognitive and clinical variables to identify those that best predicted HRQoL.

The pattern of cognitive deficits, sleep disturbances, mood alterations and impulsivity observed in the AUD patients included in this study is in accordance with the literature. Indeed, cognitive impairments have repeatedly been found in recently detoxified AUD patients [37], with deficits of verbal episodic memory, executive functions, processing speed and working memory [38–40]. Sleep disturbances are also frequently reported by patients [41]. Moreover, AUD is often associated with mood disorders [42]. Finally, sAUD patients exhibited more traits of impulsivity than controls, in line with several reports [43].

It is well known that AUD patients suffer from a poor QoL/HRQoL [11] for a review). In the present study, we used a tool specifically designed for this clinical population and found that recently detoxified sAUD patients were able to report the harmful effects their severe and chronic alcohol consumption had on different domains of HRQoL. Previous studies have shown that some demographic features of AUD patients are significantly associated with QoL. Thus, increasing age [20], being a woman [20, 23, 44, 45], having a low education level [45] and living alone [46] have a negative impact on QoL. However, in agreement with other studies that did not show any association between demographic variables and QoL (see for example [47] ), we did not find such relationships in our sample of sAUD patients when considering HRQoL specifically.

Our analyses did not reveal any significant relationship between cognitive abilities and HRQoL either. This result stands at odds with other studies showing a link between cognitive functioning and QoL/HRQoL in various conditions such as Alzheimer’s disease and Mild Cognitive Impairment [48], multiple sclerosis, stroke, and Huntington’s disease [49]. Our findings suggest that the cognitive tasks we used are either not sensitive enough to reveal strong associations with HRQoL or do not target the relevant cognitive abilities. As emotion decoding and social cognition are known to be impaired in AUD patients [50] and to affect interpersonal relationships [51], further studies assessing these functions are needed to unravel the determinants of HRQoL. Another explanation of this absence of relationship between cognitive abilities and HRQoL could be that AQoLS is a self-reported questionnaire potentially affected by cognitive deficits or self-awareness impairments [39]. It would thus be difficult to find associations between objective cognitive measures and subjective self-evaluation. However, as previously mentioned, such relationships have been found in other clinical populations with cognitive impairments and we did not systematically find significant relationships between subjective measures. Indeed, contrary to our expectations, we did not observe any association between subjective sleep quality and self-assessment of HRQoL in the present study. Nevertheless, the deleterious effect of sleep disturbances on general QoL has been revealed in dementia [52], epilepsy and multiple sclerosis [53, 54], and psychiatric disorders [55]. In AUD, a few studies reported that poor sleep quality is related to poor QoL [42] or is an important component of HRQoL [56]. The absence of relationship between sleep quality and HRQoL in our study could be explained by the fact that recently detoxified sAUD patients with executive deficits could not be cognitively able to accurately self-evaluate their sleep [57]. Further investigations using objective sleep measures are required to better understand the relationship between sleep and HRQoL in AUD.

Several studies have addressed the relationships between mood and QoL/HRQoL in AUD [58]. Both depression and anxiety may account for impaired QoL in AUD patients [23, 59]. In agreement with these studies, we found a significant association between mood variables (depression/ anxiety) and HRQoL. Thus, the more sAUD patients express symptoms of depression and anxiety, the lower was their HRQoL.

In contrast with previous studies conducted in AUD and binge drinking, we did not find any significant correlation between HRQoL and the AUDIT score, the daily alcohol consumption during the month preceding withdrawal, or the age of onset of AUD [60] [61]. This absence of relationship suggests either that the severity of AUD is not related to HRQoL or that quantitative measures of alcohol history used in the present study are not good indicators. Craving intensity or psychological dependence may be better determinants of HRQoL and should be further investigated.

We identified impulsivity and anxiety, among the studied variables, as the best determinants of HRQoL in AUD: the more sAUD patients were anxious and impulsive, the poorer was their HRQoL. The exploratory analysis showed that “control”, “activities”, “self-esteem” and “sleep” domains were especially impacted by anxiety and impulsivity. We also showed that the “living conditions” domain was impacted by anxiety, as well as the “negative emotions” domain was impacted by impulsivity.

The fact that anxiety and impulsivity were the only significant determinants of HRQoL in AUD is clinically highly relevant. Several studies have found that anxiety is a significant determinant for QoL in AUD (see for example [62]). Indeed, anxiety may have a bidirectional relationship with AUD. Indeed, some people with anxiety disorders tend to use alcohol as a self-medication to help coping with their symptoms, which in turn favor the development of AUD [63, 64]. Conversely, other studies have shown that AUD also induces anxiety symptoms [65, 66, 67]. Anxiety appears therefore as a critical factor to consider in the treatment of AUD patients.

Impulsivity has been found to impair QoL in patients with compulsive behaviors [68] or bipolar disorder [69]. Impulsivity results in serious physical, social and psychological issues, which would lead in cascade to functional impairments. In bipolar patients, it was found that impulsivity influences QoL both indirectly through altered level of functioning and more directly as suggested by the relationship between impulsivity and QoL that remains significant after controlling for the level of functioning [70]. The fact that impulsivity was an important determinant of HRQoL in AUD is clinically highly relevant. Indeed, several studies indicated that impulsivity may not only precede the development of AUD [71] but also favors relapse [72] even after sustained abstinence [73].

In AUD, taking account of anxiety and impulsivity is thus crucial since they contribute, notably in an indirect way through alteration of HRQoL, to the treatment outcome. In this respect, in addition to pharmacological treatments, several interventions such as physical activity [74] or meditation [75] could be proposed to manage anxiety and impulsivity in AUD.

### Limitations

It is worthwhile noting that AQoLS has been validated only with exploratory factorial analysis and calculation of internal consistency using the Cronbach alpha. While this method has been for long widely accepted, it is no more considered as a satisfactory one. The sample size of the present study does not allow us to report additional data on the validity and feasibility of the measure. Additional studies on a large cohort of patients are needed to fully validate this tool.

## Conclusion

To conclude, our study is the first to assess HRQoL in a group of carefully selected recently detoxified sAUD patients using a questionnaire especially designed for this clinical population, combined with a neuropsychological examination, assessments of subjective sleep quality, mood, impulsivity, and alcohol history indicators. Our findings reveal that in sAUD patients, anxiety and impulsivity are crucial determinants of HRQoL. Thus, anxiety and impulsivity should be more systematically investigated and targeted by non-pharmacological interventions in order to improve treatment outcomes.

## Data Availability

The datasets used and/or analysed during the current study are available from the corresponding author on reasonable request.
